# Porous Organic Polymers: An Emerged Platform for Photocatalytic Water Splitting

**DOI:** 10.3389/fchem.2018.00592

**Published:** 2018-12-04

**Authors:** Chen Xu, Weijie Zhang, Juntao Tang, Chunyue Pan, Guipeng Yu

**Affiliations:** Hunan Provincial Key Laboratory of Efficient and Clean Utilization of Manganese Resources, College of Chemistry and Chemical Engineering, Central South University, Changsha, China

**Keywords:** porous organic polymers, heterogeneous catalysis, photocatalyst, water splitting, oxygen evolution, hydrogen evolution

## Abstract

Porous organic polymers (POPs), known for its high surface area and abundant porosity, can be easily designed and constructed at the molecular level. The POPs offer confined molecular spaces for the interplay of photons, excitons, electrons and holes, therefore featuring great potential in catalysis. In this review, a brief summary on the recent development of some current state-of-the-art POPs for photocatalytic water splitting and their design principles and synthetic strategies as well as relationship between structure and photocatalytic hydrogen or oxygen evolution performance are presented. Future prospects including research directions are also proposed, which may provide insights for developing POPs for photocatalytic water splitting with our expectations.

## Introduction

**Figure 1:**
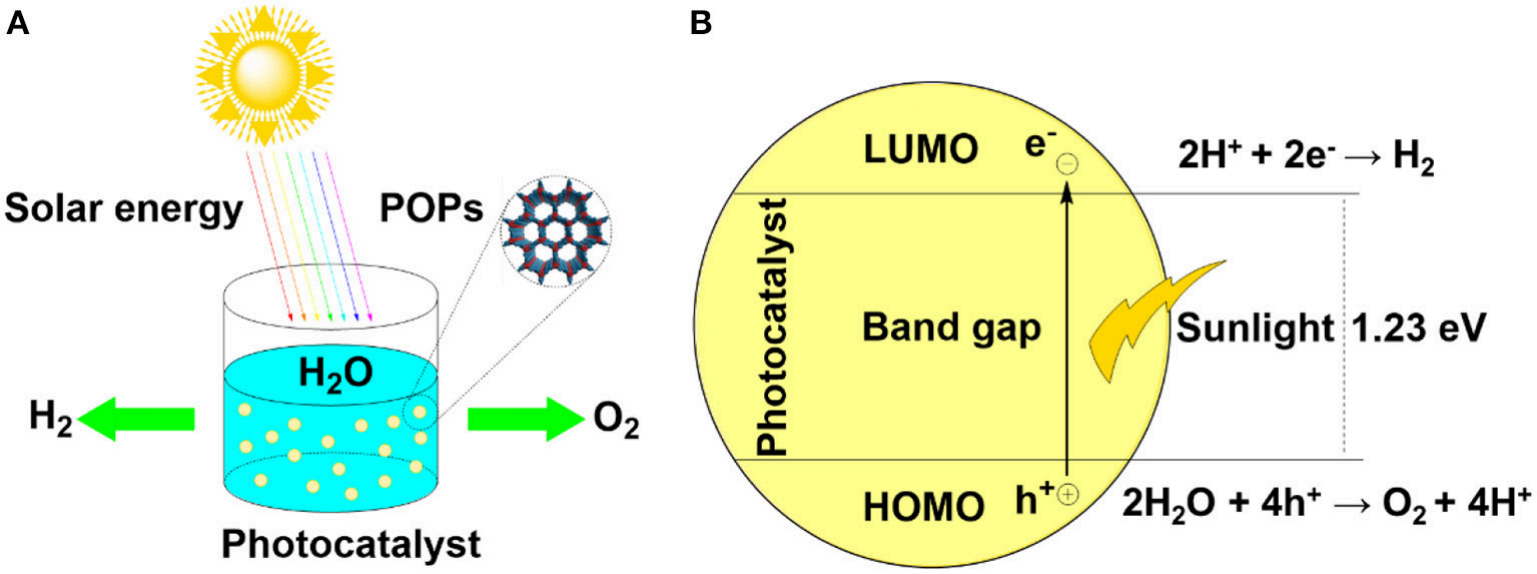
**(A)** Schematic diagram of photocatalytic water splitting, inset is a skeleton of POPs **(B)** Scheme of basic processes in photocatalytic water splitting using semiconductors.

Visible-light-driven water splitting via photocatalysis has witnessed explosive advances in the past decade, accompanying the enormous growth of the photocatalyst design. Among the intensely investigated systems, porous organic polymers (POPs) have demonstrated their ability to be a versatile platform for water splitting (Figure 1A). POPs are a kind of covalently connected polymers with high specific surface area and permanent, nanometer-scale pores which are beneficial to exposing active sites for catalytic conversions (Zhang and Riduan, 2012). Unlike the other porous counterparts such as zeolites, silicas, metal–organic frameworks (MOFs), metal–organic cages, POPs are designable; that is, the nature of building blocks including geometry and dimensions can be controlled targeting different functions. Besides, POPs connected through strong covalent linkages always feature high chemical and thermal stability, which is a prerequisite for recyclable photocatalytic application. In general, typical POPs mainly include conjugated microporous polymers (CMPs) (Jiang et al., 2008; Cooper, 2009; Schmidt et al., 2009), covalent triazine frameworks (CTFs) (Kuhn et al., 2008; Bojdys et al., 2010), and covalent organic frameworks (COFs) (Cote, 2005; Tilford et al., 2006; Ding and Wang, 2013; Huang et al., 2016).

Given the high-throughput research efforts on photocatalytic hydrogen evolution reaction (HER), Wang et al summarized the progress on conjugated polymer catalysts for HER in 2016 (Zhang et al., 2016). However, boosting works on this field have been disclosed and the researches on photocatalytic oxygen evolution reaction (OER) also have gained fundamental understanding lately. Therefore, it is essential to give another review about the latest developments. Herein we summarize the synthetic strategy, molecular design, and functional modulation of CMPs, COFs and CTF, and more importantly highlight their performance for water splitting.

## Basic Principles of Photocatalytic Water Splitting

From a fundamental perspective, overall water splitting into hydrogen and oxygen requires a change on the standard Gibbs free energy of ΔG^0^ of 237 kJ/mol (**Equation 1**) (Ananyev and Dismukes, 2005). The minimum band gap could be calculated by assuming that the water splitting energy was entirely converted from electrical work (**Equation 2**). In other words, the band gap should be over 1.23 eV to meet the requirement of thermodynamics of water splitting when the semiconductors are used as photocatalysts. Meanwhile, the band gap of visible light response-semiconductors should be lower than 3.0 eV (**Equation 3**), since broader band gap would result in a low efficiency in utilization of the visible light (>400 nm).

(1) $$\begin{array}{rcl} {\text{H}}_{2}O\to {\text{H}}_{2}+1/2{\text{O}}_{2},\;△{\text{G}}^{0} & = & 237\text{kJ}/\text{mol} \end{array}$$

(2) $$\begin{array}{rcl} △{\text{G}}^{0} & = & {\text{W}}_{\text{f},\text{max}}=-\text{nEF} \end{array}$$

(3) $$\begin{array}{rcl} \text{E} & = & \text{h}\nu =\text{hc}/\lambda \end{array}$$

From aspect of specific processes, when semiconductors are stimulated by the photons, the electrons in the HOMO are placidly transited to the LUMO by overcoming the hindrance of the band gap (Figure 1B). In this process, the hole (h^+^) would be retained in the HOMO while the electron (e^−^) emerges in the LUMO. In the “photo-excited” state, most of electron-hole pairs are rapidly combined. Part of the electron-hole pairs migrate to the surface and would be captured by water, causing the effective water splitting. For porous catalysts, the high surface area and well-defined pore structure would effectively facilitate the exposure of available surface electron-hole pairs and are beneficial for transmission of reactants. Therefore, three key processes are vital for water splitting. First, capturing of photons, then separation and transportation of electron-hole pairs, and water splitting by electron-hole pairs.

Sacrificial agents and co-catalyst are generally used in most situations to improve the water splitting efficiency of an organic and polymeric photocatalyst system. Sacrificial agents were used to prohibit undesirable charge recombination. In other words, these sacrificial agents can quickly capture the photoexcited holes or electrons, and therefore promote the transformation. In typical HER processes, methanol, triethanolamine (TEOA) and lactic acid etc are usually used as sacrificial agents of holes, which are easier to be oxidized than water. Similarly, in OER process, silver nitrate (AgNO_3_) and sodium iodate (NaI) are used as sacrificial agents of electrons to accelerate water oxidation. Co-catalyst is cooperated to the photocatalytic system to reduce the potentials of hydrogen reduction and provide more active sites. Besides, the co-catalyst can promote the separation of the photoexcited electron-hole pairs on the photocatalyst surface, leading to an enhanced photocatalytic system. In a general HER, H_2_PtCl_6_ is loaded on the surface of polymers by photo-deposition method (Kaur et al., 2011; Ou et al., 2017). In addition, the transition metals in VIII clan such as Ru (Wang et al., 2009), Ni (Chang et al., 2015), Au and Ag (Paramasivam et al., 2008) can also be used as co-catalysts for photocatalytic water splitting.

When we assess a photocatalyst of water splitting, the rate of hydrogen or oxygen evolution is the most commonly used indicator of photocatalytic activity. However, the rates of evolution are susceptible to photodynamic conditions. Apparent quantum yield (AQY) that represents the efficiency of the photocatalytic process is more sensible for comparison (Zhang et al., 2016), but unfortunately absent in many studies. The AQY is calculated based on the equation (Zhang et al., 2013) as follows:

(4) $$\begin{array}{lll} \text{AQY}\left(\%\right) & = & \frac{\text{number of reacted electrons}}{\text{number of incident photons}}\times 100\\ = & \frac{2\times \text{number of evolved }{\text{H}}_{2}\;molecules}{\text{number of incident photons}}\times 100\\ = & \frac{4\times \text{number of evolved }{\text{O}}_{2}\;molecules}{\text{number of incident photons}}\times 100 \end{array}$$

The stability of photocatalysts that determined by long time experiment or repeat experiment is indispensable. Generally, POP photocatalysts are durable relative to their counterparts, and only slight weakened activities that mainly cause by the inevitable photocorrosion. Other factors such as light absorbance, surface area, morphology, energy band and charge carrier separation and transport abilities may also exert an effect on the photocatalytic water splitting. Based on these parameters, we overviewed a range of structurally diversified POPs, and gave an explicit comparison of their photocatalytic activities for water splitting.

## POPs for Water Splitting

### Conjugated Microporous Polymers

**Figure 2:**
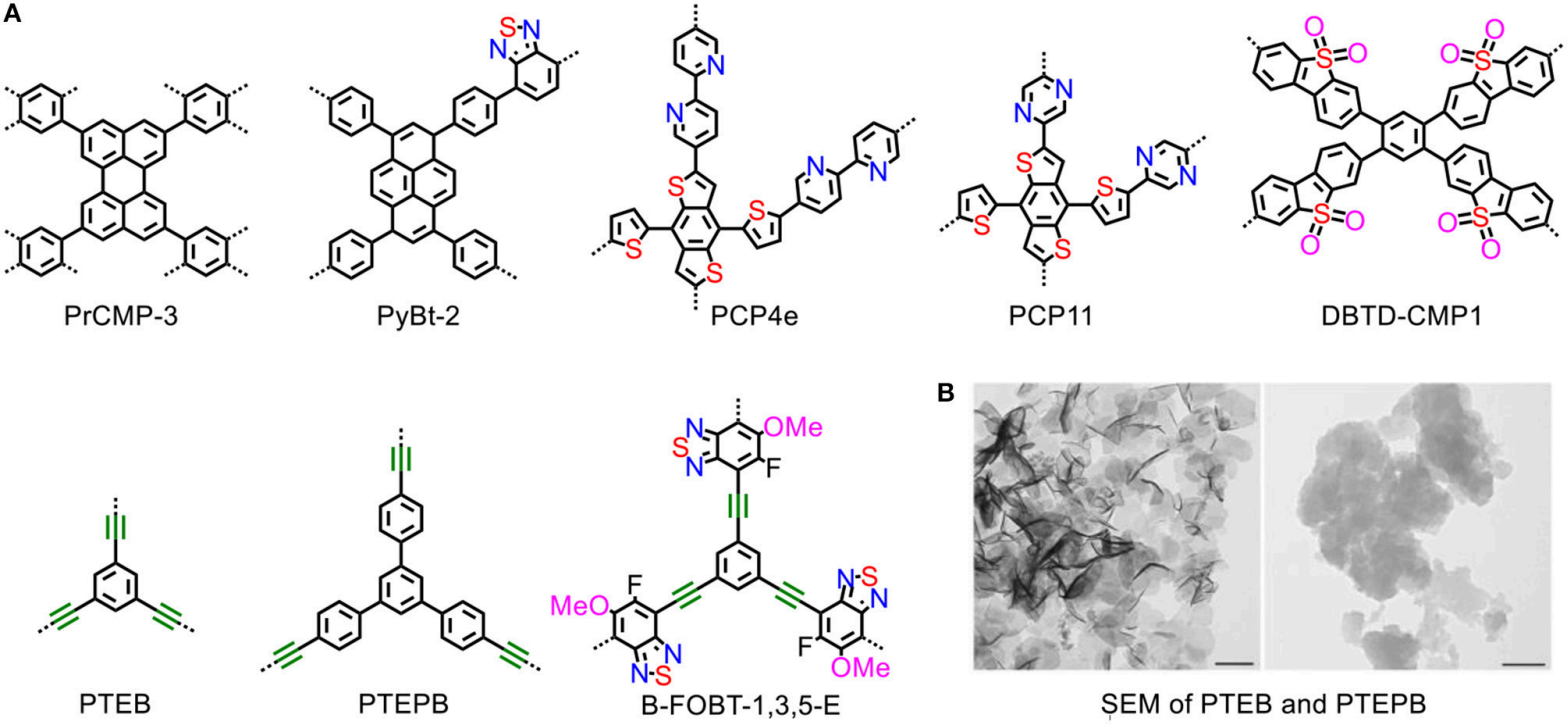
**(A)** Structures of the CMPs. **(B)** SEM images of sheet-like morphology, the scale bars are 200 nm (Wang et al., 2017b). Reproduced with Permission from Wiley-VCH.

Conjugated microporous polymers (CMPs) are comprised of π-conjugated scaffold and large electron delocalized network structure, which would be in favor of efficient light absorption and charge carrier transportation (Figure 2A). Various design strategies have been presented to improve the photocatalytic activities of CMPs (Li et al., 2016a,c,d).

Pyrene and perylene are typical electron-rich units with unique optical activities such as high light-absorption coefficient and electron donating ability. Pioneering pyrene-based donor-π (D-π) CMPs for photocatalytic water splitting were proposed (Sprick et al., 2015). However, the hydrogen evolution rate of bare powder is rather low [174 μmol/(h g)] under visible light irradiation. Jiang and co-workers developed a series of perylene-based CMPs with analogical D-π molecular structures, while the hydrogen production rate is still unattractive with a value to 13 μmol/(h g) (PrCMP-3) (Xu et al., 2017). These results demonstrate that the photocatalytic performance of full donor based CMPs are still not satisfied due to the lack of internal polarization for effective charge separation process. Therefore, there is still much room for improvement and requires further rationally designing pyrene or perylene-based CMPs.

Bearing this in the mind, Jiang's group developed a series of acceptor contained pyrene-based CMPs by introducing different percentile electron-withdrawing benzothiadiazole into the skeleton. To note that the most active photocatalyst of PyBt-2 derived from copolymerization of donor units (i.e., 1,3,6,8-tetrabromopyrene) and accepter monomers(i.e., 4,7-dibromobenzo[c][1,2,5]thiadiazol) through a π linkage of 1,4-phenylene, which shows significantly enhanced photocatalytic activity for visible-light driven hydrogen evolution at a rate of 296 μmol/(h g) and considerable activity of OER (Xu et al., 2018). Clearly, D-A conjugated CMPs exhibit exceptional photocatalytic activities that can be finely tuned by adjusting the ratio of donor and acceptor units. When polarized skeleton favors the separation of electron-hole pairs, and fully conjugated skeleton favors the charges transport. There is still a “balance” in the competition of these two contributions, which requires a proper ratio of donor and aci-cepter. Besides, the sequence of polymer chain remains chaos while considering the different reactivity of comonomers.

Recently, more CMPs–based photocatalysts are rationally designed through the copolymerization of various ratios of photovoltaic donor and acceptor comonomers. Notably, the CMP derived from 4,8-di(thiophen-2-yl)benzo[1,2-b:4,5b']dithiophene chromophore (DBD) shows a significantly high hydrogen production rate up to about 1,914 μmol/(h·g) (Li et al., 2016a). The incorporation of nitrogen-doped acceptor ligand such as pyrazine and dipyridyl was also proved effectively to enhance the photocatalytic activity, and the hydrogen evolution rate is up to 2,590 μmol/(h·g) (Li et al., 2016d). The CMPs containing dibenzothiophene dioxide (DBTD) exhibit an exceptionally high rate up to 4,600 μmol/(h·g). Besides, DBTD-CMP-1 was demonstrated as an efficient photocatalyst for cocatalyst-free HER that produces hydrogen at rate of 2,460 μmol/(h·g) under visible-light irradiation (Wang et al., 2018). Above all, numerous units with specific electronic properties and geometry are introduced into the backbone of the D-A CMP catalytic system, and the visible-light driven hydrogen evolution rate is accelerated significantly.

The introduction of alkynyl groups into the conjugated polymers is an effective and facile strategy for developing photocatalyst for water splitting. Enhancement of photocurrent intensity and negative shift of LUMO levels are caused by the presence of the alkynyl groups, and improve visible-light-drived H_2_ production activity (Zhang et al., 2017b). The alkynyl-based CMPs obtained from the oxidation coupling of 1,3,5-triethynylbenzene (TEB) and 1,3,5-tris-(4-ethynylphenyl)-benzene (TEPB) can split water into stoichiometric hydrogen and oxygen at moderate evolution rates (218 and 102 μmol/(h·g) with high AQYs for overall water splitting (10.3 and 7.6%) under visible-light irradiation. In the study, the sheet-like morphology of polymers may enable the photogenerated excitons to be easy to access the polymer surface to drive redox reactions (Figure 2B), suppressing undesirable electron–hole recombination (Wang et al., 2017b). Similarly, alkynyl-based B-FOBT-1,3,5 with D-A conjugated structures show very impressive activity at a hydrogen evolution rate of 9,600 μmol/(h·g), and this enhancement is contributed by the D-A structure which facilitates proton-coupled electron transfer (PCET) process (Xiang et al., 2018).

The photocatalytic CMPs are facile to integrate with various monomers and optimize with optional ratio of different monomers via Suzuki-coupling, oxidative coupling and Sonogashira coupling etc. The copolymerization extends delocalized π-conjugation that is advantageous for harvest of photons, and the tunable polarized skeleton offers high efficient separation of electron-hole pairs. Briefly, CMPs are fully accessible to the box of photoactive units for creation of high-performance D-A skeleton, and this advantage makes CMP a frontier material with high activity on water splitting.

#### Covalent Triazine Frameworks

Covalent triazine frameworks (CTFs) which feature high thermal and chemical stability, were generally constructed from triazine precursors or aromatic nitriles. From a chemical view, triazine known as a nitrogen-rich conjugated unit with electron withdrawing character may act as the redox active site for photocatalytic water splitting (Schwinghammer et al., 2013, 2014; Stegbauer et al., 2014). Given these properties, CTFs are widely employed as heterogeneous catalysts (Puthiaraj et al., 2016) and have shown attracting photocatalytic activities under visible light irradiation (Li et al., 2016b; Qu et al., 2016; Meier et al., 2017).

**Figure 3:**
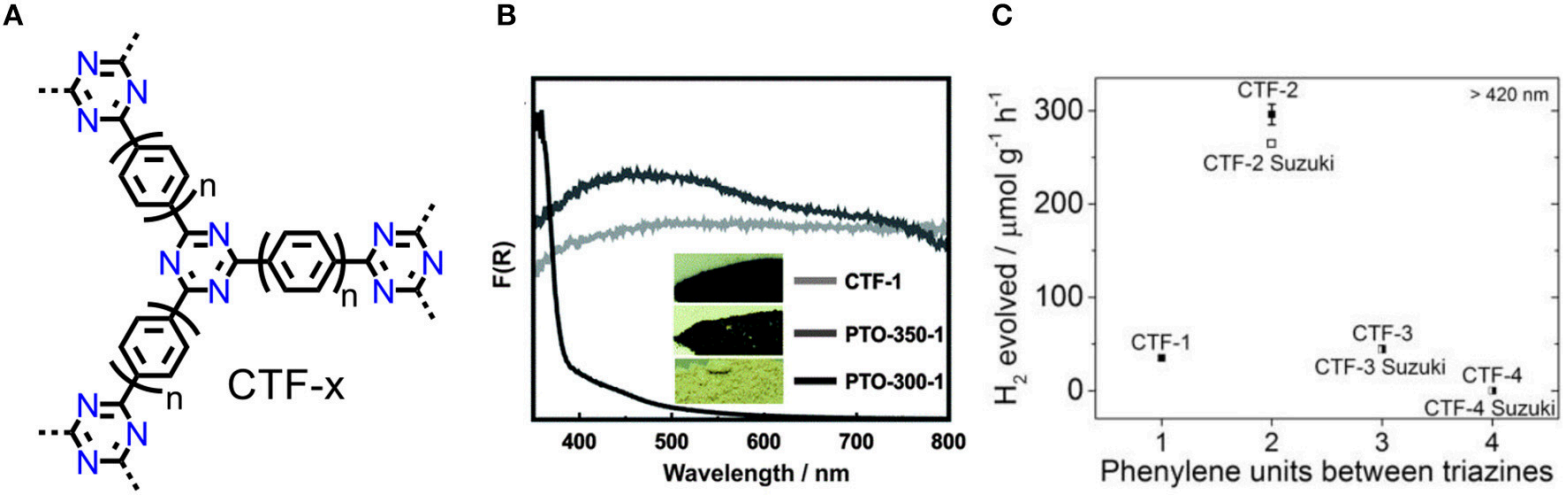
**(A)** Notional structures of CTFs (CTF-1: *n* = 1; CTF-2: *n* = 2; CTF-3: *n* = 3. CTF-4: *n* = 4). **(B)** UV/vis absorptions and photographs (inset) of PTO-300, PTO-350, and CTF-1. (Schwinghammer et al., 2015). Reproduced with Permission from The Royal Society of Chemistry. **(C)** HER compare of CTF-1~ CTF-4 and Suzuki coupled CTF-2~ CTF-4 (Meier et al., 2017). Reproduced with Permission from Elsevier.

CTF-1 was prepared from terephthalonitrile via an ionothermal method as a black monolithic powder (Figure 3A; Kuhn et al., 2008), which lacks sufficient thermodynamic driving force for photocatalytic water splitting. The reason may be that the partial carbonization of CTFs, then results in the narrowed band gap. Bearing this in mind, Lotsch et al optimized the ionothermal method using of low reaction temperature and long reaction time. The Pale yellow PTO-300 sample was obtained and shows enhanced HER rate (up to 1,000 μmol/(h·g)) under simulated sunlight irradiation (Figure 3B) (Schwinghammer et al., 2015). Therefore, the appropriate band gap is prerequisite to photocatalytic hydrogen production after avoiding unexpected carbonization. PTO-300 is of low degree for polymerization and insufficient delocalized conjugated system, hence it can be expected that novel synthesis strategies of CTFs may lead to further developments of photocatalysis.

Latterly, more methods for constructing CTFs were proposed such as microwave assist-ionothermal method (Zhang et al., 2010a) and the use of strong Brønsted acid (Ren et al., 2012; Zhu et al., 2012). CTF-T1 was obtained from trifluoromethanesulfonic acid (TfOH) catalyzed trimerization of terephthalonitrile at room temperature, and gives an acceptable band gap of 2.94 eV (Bi et al., 2015). HER rate is competitive to g-C_3_N_4_ (Wang et al., 2009) and titanium dioxide nanocrystals (Chen et al., 2011), and considerably higher than ionothermal obtained triazine-based oligomers (Schwinghammer et al., 2015), the AQY of CTF-T1 is about 2.4% at 420 nm. Photocatalytic OER of CTF-T1 was also tested, however, the evolution rate is only 9 μmol/(h·g). Cooper et al synthesized a series of CTFs from CTF-1 to CTF-4, either by TfOH catalyzed trimerization of nitriles or by Suzuki-Miyaura polymerization (Figure 3A; Meier et al., 2017). The obtained CTFs show different band gaps with those of CTFs from Suzuki-Miyaura coupling, which is originated from the difference on the end-group nature and the defects in the materials. CTF-2 shows the highest HER (296 μmol/(h·g)) among the as-made materials while the HER of CTF-2-Suzuki is lower due to its low surface area (Figure 3C). It is attracting that the extension of phenylene spacer reduces thermodynamic driving force for water splitting but simultaneously broaden the visible-light absorbance. The photocatalytic activity of CTF photocatalytic system can be facilely regulated by a synergetic effect of light absorbance, band gap and surface area.

The next generation of porous materials, however, already seems to be underway, as represented by “porous photocatalysts for oxygen evolution” (Garcia-Esparza et al., 2017; Wang et al., 2017c; Zhang et al., 2017a). Considering the easy tailorable building blocks, CTF gives a synthetically versatile platform for photocatalytic water splitting. The nitrogen-rich framework provides active site for doping and coordination site for modification with metal-photocatalyst that facilitate to enhance the activity of CTF. Over the last couple of years, CTF attracts colossal interests by its extremely high activity on oxygen evolution. The narrow band gap and deep HOMO have indicate the strong oxidative activity of CTF, and a promising photocatalyst for water oxidation.

**Figure 4:**
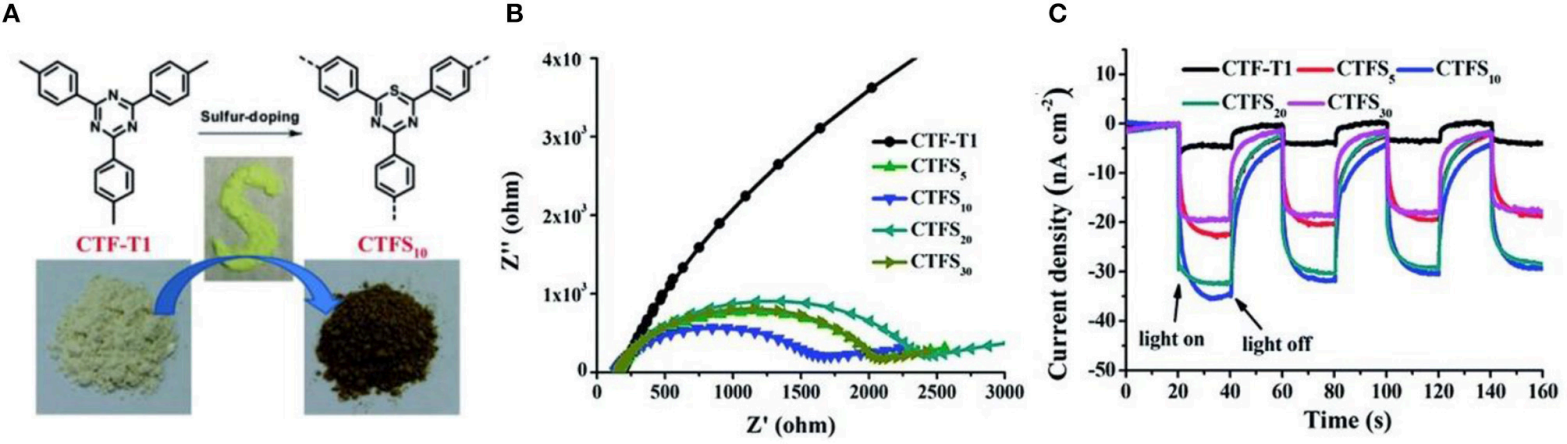
**(A)** Scheme of sulfur doping method on CTFs **(B)** EIS Nyquist plots and **(C)** Photocurrent response of CTF-T1 and CTFS (Li et al., 2016b). Reproduced with Permission from The Royal Society of Chemistry.

Typically, Wang et al described the preparation of cobalt-modified CTP-2 through trimerizations of 4,4'-biphenyldicarbonitrile and it shows the considerable photocatalytic activity of OER at a rate of 30 μmol/(h·g) under visible light irradiation (Lan et al., 2018). Moreover, a crystalline CTF-1-100W was prepared under mild microwave-assistance in the presence of TfOH, and the ordered in-plane skeleton has been retained with minimal carbonization. With a sufficient harvest of visible-light photon, appropriate energy band and low HOMO are the dominant factors for OER, and highest AQY (3.8% at 420 nm) of photocatalytic oxygen evolution is achieved. Remarkably, the CTF-1-100W is also efficient for HER with evolution rate of 5,500 μmol/(h·g) and AQY of 6% under visible-light irradiation (Xie et al., 2018). The photocatalytic activities can be significantly improved by doping methods (Figure 4A; Zhang et al., 2014; Ran et al., 2015). Li et al developed a sulfur-doped CTFS_10_ by annealing treatment with sulfur (Li et al., 2016b), and the rate of HER is estimated at 2,000 μmol/(h·g), which is four times higher than CTF-T1. The CTFS show smaller impedance of charge (Figure 4B) and lower intensity of photoexcited luminescence (Figure 4C) than CTF-T1, since introduction of electron-rich sulfur promotes the coupling between the triazine ring and the phenyl rings and impedes the recombination of photoexcited electron-hole pairs.

The modification of chemical composition and physical structure is a prospective strategy to enhance the activity of organic photocatalysts (Sun and Liang, 2017). The MoS_2_ quantum dots (QDs) was modified on CTF-T1 via the *in situ* photo-deposition method, which endow the synergistic composite an 8 times higher rate of HER than the pure CTF-T1 (Jiang et al., 2018). The strong electron interaction between MoS_2_ and CTF-T1 is a possible result from the formation of heterojunction (Christoforidis and Fornasiero, 2017), However, further investigation is still needed on these composite photocatalysts and the detailed mechanism remains unclear.

Most CTFs with good photocatalytic activity are amorphous in nature. The crystallinity facilitates the expose of photocatalytic activities of polymeric semiconductor (Wang et al., 2012; Zhang et al., 2015) due to their open-pore arrays. The strong Brønsted acid catalyst was used to reduce the carbonization, while damages the crystallinity of CTFs. Recently some novel synthesis method has been proposed such as low temperature polycondensation approaches (Wang et al., 2017a; Yu et al., 2018). The crystalline CTFs obtained by these mild approaches may show great potentials in photocatalytic water splitting. In addition, structural design and the synthetic process still challenge for CTFs, and more attentions still need to be paid to clarifying the relationships between its structure and photocatalytic properties.

#### Covalent Organic Frameworks

Covalent organic frameworks (COFs) represent a high crystalline type of POPs (Huang et al., 2016), which are precisely integrated into extended periodic skeletons via reverible condensation reaction such as Schiff base reaction (Cote, 2005). In comparison with other POPs, COFs featured attracting uniform porous structure and high ordered skeleton (Ding and Wang, 2013).

**Figure 5:**
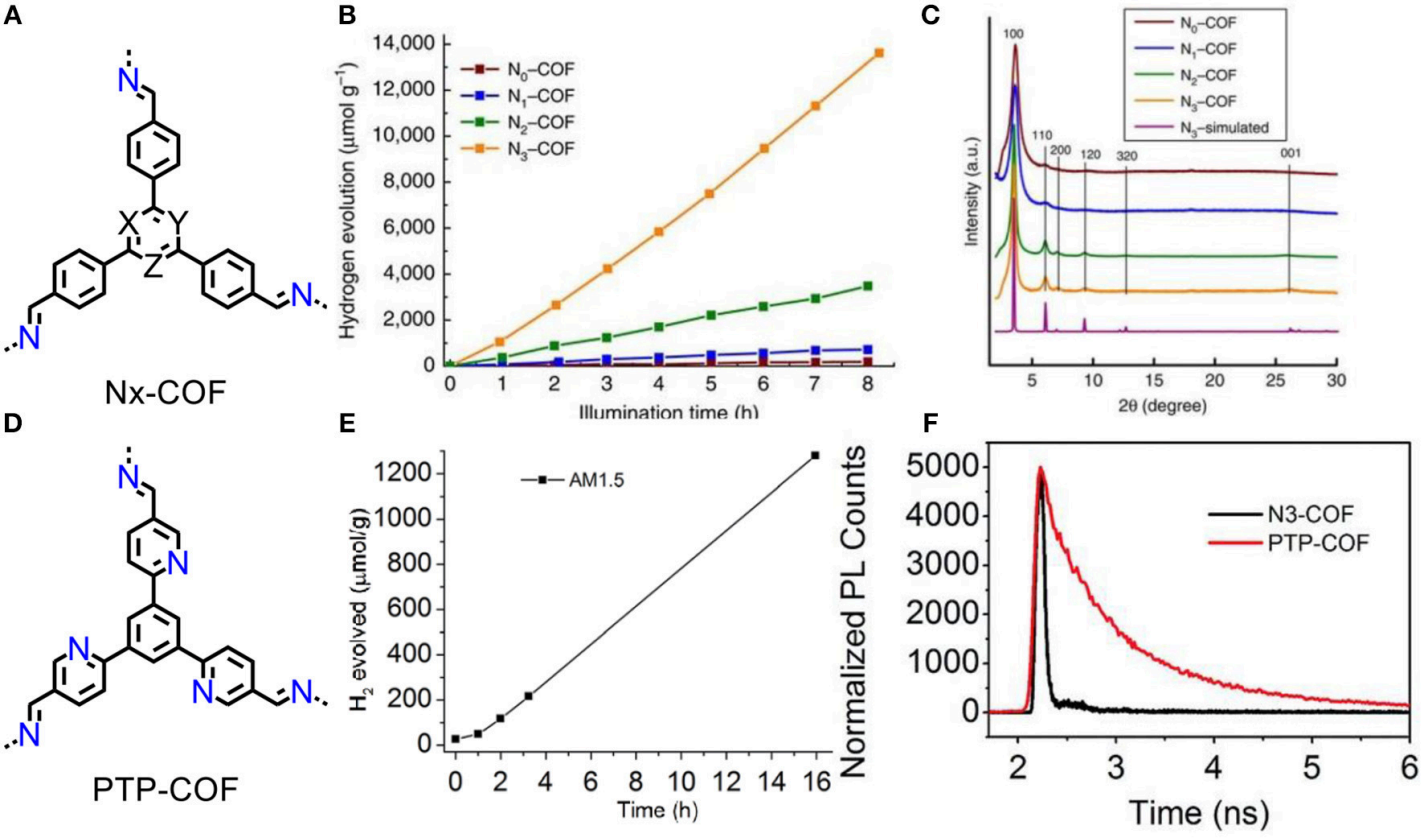
**(A)** Structures of the Nx-COFs (X = Y = Z = C-H: N0-COF; X = N, Y = Z = C-H: N1-COF; X = Y = N, Z = C-H: N2-COF; X = Y = Z = N: N3-COF). PXRD patterns **(B)** and hydrogen evolution rates **(C)** of N0-COF to N3-COF (Vyas et al., 2015). Reproduced with Permission from Nature Publishing Group **(D)** Structures of the PTP-COF. Hydrogen evolution rates **(E)** and time correlated single-photon counting (TCSPC) decay trace **(F)** of PTP-COF (Haase et al., 2017). Reproduced with Permission from The Royal Society of Chemistry.

Recently, Lotsch and co-workers developed a series of azine COFs (Figure 5A) (Vyas et al., 2015) and the resultant N3-COF exhibits a hydrogen evolution rate at 1,703 μmol/(h·g) (Figure 5B). The activities of N1-COF~N3-COFs are positively correlated with crystallinity that increase along the series (Figure 5C). In order to further verify how the nitrogen content influence the activity of this photocatalytic system, another PTP-COF was also investigated (Figure 5D) (Haase et al., 2017). The nitrogen atoms in peripherals lead to irregular stacking of frameworks that causing a poor crystallinity, thus HER rate is sharp decrease to 84 μmol/(h·g) which an order of magnitude lower than the N3-COF (Figure 5E). As the unsatisfied activity of PTP-COF, it has more fluorescence-consuming recombination of electron-hole pairs than N3-COF, which are evidenced by the fluorescence decay (Figure 5F). Substitution of C-H units with N atoms on central aryl ring decreased the dihedral angle between the central aryl ring and the peripheral phenyl ring in the COF nodes, thus the enhanced activity is dominated by the increase in planarity of conjugated skeleton and crystallinity of material. The high planarity of conjugation increases charge carrier mobility, and crystallinity of COFs facilitates to prevent photoexcited electrons-holes recombination and decrease the trapping at defect sites.

**Figure 6:**
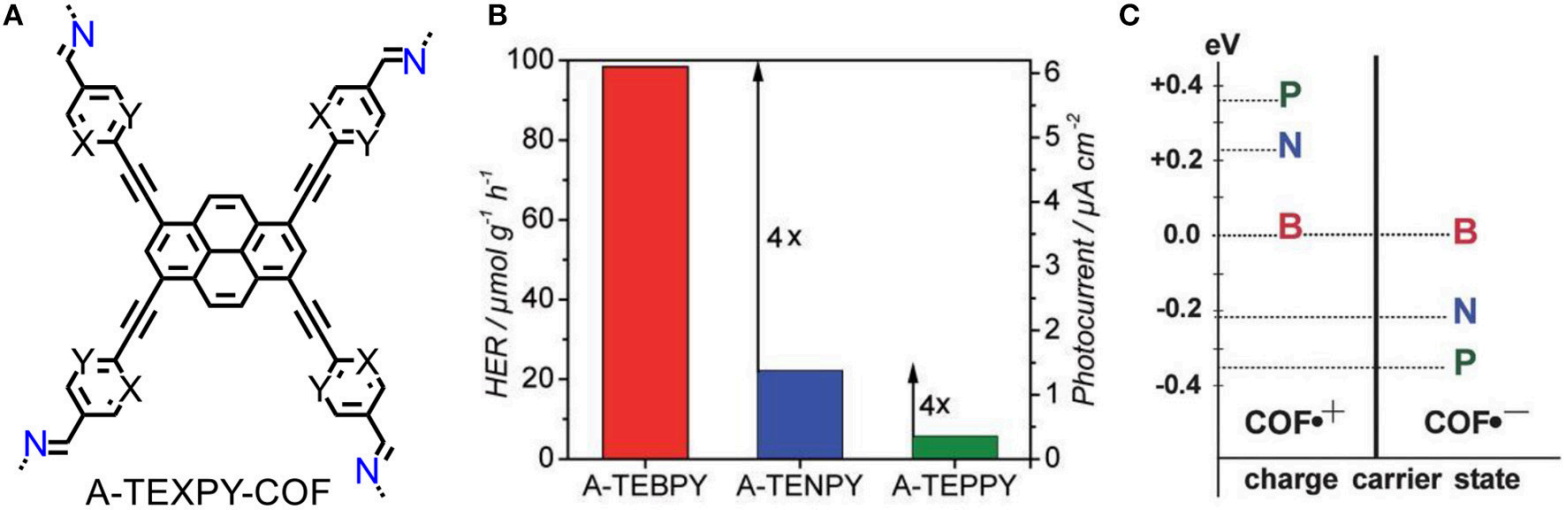
**(A)** Structures of the A-TEXPY-COFs (X = Y = C-H: A-TEBPY-COF; X = N, Y = C-H: A-TENPY-COF; X = Y = N: A-TEPPY-COF). **(B)** Hydrogen evolution rates of A-TEXPY- COFs. **(C)** Comparison of vertical radical cation and radical anion stabilization energies relative to the A-TEBPY COF system (Stegbauer et al., 2018). Reproduced with Permission from Wiley.

Three azine-linked COFs were constructed with pyrene building blocks that vary in number of substituted nitrogen atoms in peripheral phenyl ring (Figure 6A) (Stegbauer et al., 2018). The photocatalytic activities of A-TEXPY-COFs are highly relevant with the stability of intermediate of water splitting. Upon increasing stabilization of radical anion that conceivable intermediate of electron-rich n-type semiconductor, the rate of hydrogen evolution is dramatically higher (Figures 6B, C). The theoretical calculation of the stabilization of intermediate could be an effective guidance for molecular designation of high-performance photocatalytic COF.

**Figure 7:**
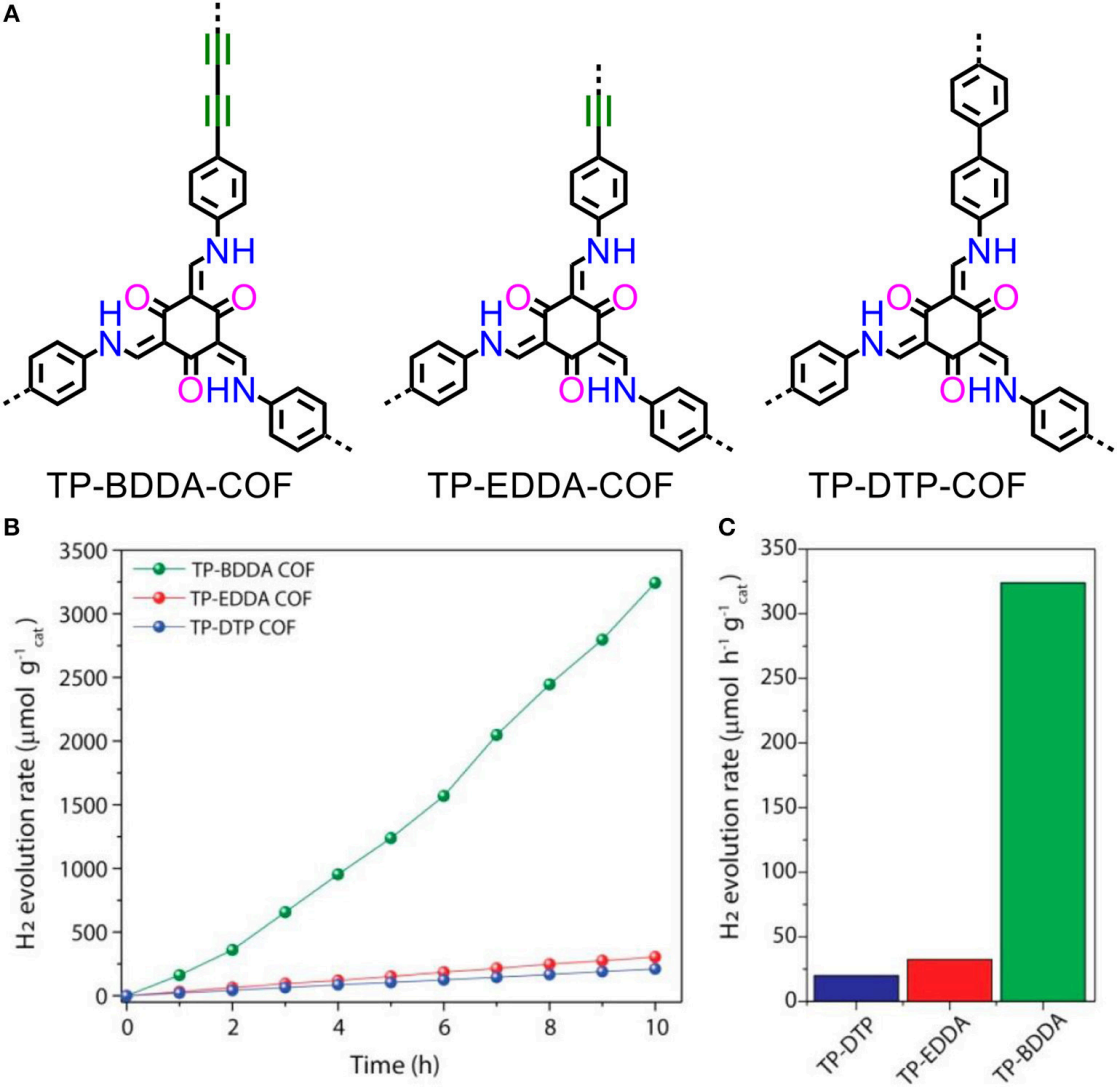
**(A)** Structures and **(B,C)** hydrogen evolution rates of TP-BDDA-COF, TP-EDDA-COF and TP-DTP-COF (Pachfule et al., 2018). Reproduced with Permission from American Chemical Society.

The β-ketoenamine linked COFs are constructed by Schiff-base condensation of alkynyl functionalized amines with 1,3,5-triformylphloroglucinol (Figure 7A). The diacetylene functionalized COF exhibits relative high rates of HER (at average 324 μmol/(h·g)) (Figures 7B, C). Most importantly, a high AQY at 1.8% was obtained under 520 nm light irradiation (Pachfule et al., 2018). Similar to the CMP, design principle that introducing alkynyl group is an efficient strategy to promote the hydrogen production activity. This reduces the LUMO and broadens the visible-light absorbance of the polymer.

**Figure 8:**
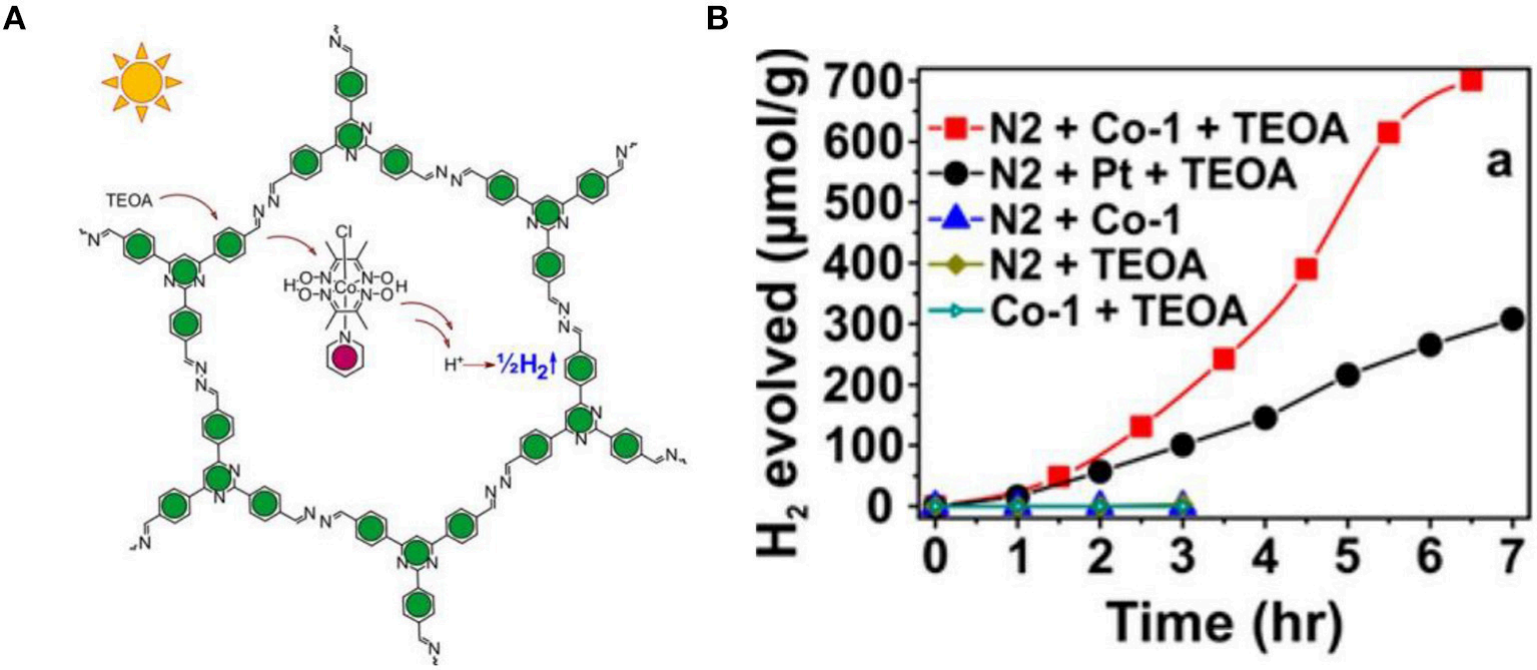
**(A)** Schematic representation of photocatalytic hydrogen evolution with N2-COF and Co-co-catalyst. **(B)** Hydrogen evolution rate of N2-COF and Cobalt co-catalyst as well as N2-COF and metallic platinum in the presence of TEOA (Banerjee et al., 2017). Reproduced with Permission from American Chemical Society.

Up to now most studies on COFs for photocatalytic hydrogen production use platinum as the co-catalyst. More recently, Lotsch and co-workers presented a noble-metal-free photocatalytic hydrogen production using N2-COF as photocatalyst (Figure 8A) (Banerjee et al., 2017). Interestingly, non-precious-metal-based cobaloxime was used as co-catalyst, and a higher hydrogen evolution rate at 782 μmol/(h·g) was achieved compared to that using platinum as co-catalyst (Figure 8B). This result is attributable to the more efficient monometallic electron transfer from COF to the cobalt co-catalyst, and cobalt is the primary active site.

COFs are supramolecular architectures as they are precise predesigned and regular integrated, and their optical and electronic properties can be easily optimized. By virtue of their explicit structure, lots of theoretical calculation has been presented to reveal the structure-properties relationship, as they accurately predict the energy band and the stability of the intermediate.

## Conclusion and Comparison

Above all, the development of POPs for photocatalytic water splitting is promising while still challenging. Rapid progress in the past 2–3 years in the design and synthesis of POPs has revealed their great potential as tailorable materials platforms for structural and functional design of high performance catalyst. This minireview has given a slight inspiration on design, synthesis and modulation of POPs for photocatalytic hydrogen production and oxygen production. Nowadays, metal oxides as photocatalyst are most extensively studied for hydrogen production (Kudo and Miseki, 2009; Chen et al., 2010), and carbon nitrogen materials are high efficiency photocatalysts which attract much attention in recent years (Lin et al., 2016; Zhang et al., 2018). As a competitor to these state-of the-art photocatalysts, plenty of well-designed POPs with diverse building blocks, synthetic methods and versatile functionality are disclosed. More specifically, CMPs are facility to processing from diverse monomers, and allow the easy introduction of task-specific functional group to modulate its photocatalytic properties. The exploration on synthetic strategies such as acid catalyzed synthesis would improve the photo catalytic activities of CTFs. Advances in the synthesis methods and the exploration of new materials would provide the potential for further developments of effective photocatalysts. More importantly, the CTF has shown good activities in the oxygen production process. Besides, structural and functional design of CTF is emerged to initiate hotspot of photocatalytic research prospectively. Noticeably, the doping and modified process with remarkable improvement indicates an effective approach to enhance the properties of CTF. The COFs have prominent activities of photocatalytic hydrogen production where ordered conjugated networks facilitate to enhance the efficiency of visible-light photocatalysis and achieve high AQY under visible light irradiation. Precisely modulations of photocatalytic COFs are being discussed, revealing the structure-properties relationship and the design-regulation strategies of COFs.

Here is a comparison of the photocatalytic water splitting processes, and most recent photocatalysis performance is encompassed (Table 1).

**Table 1:** Photocatalytic hydrogen and oxygen evolution activities of POPs.

**POPs**	**Cocatalyst**	**Sacrificial agent**	**Solvent**	**Filter cut**	**Rate of HER or OER μmol/(h·g)**	**AQY**	**References**
PrCMP-3	No	20%TEOA	water	>420 nm	13		Xu et al., 2017
PyBT-2	3 wt% Pt	20%TEOA	water	>420 nm	296	0.23% at 420 nm	Xu et al., 2018
PCP4e	2 wt% Pt	20%TEOA	water	>400 nm	1,914	0.28% at 400 nm	Li et al., 2016a
PCP11	2 wt% Pt	20%TEA	water	>400 nm	2,590	1.93% at 400 nm	Li et al., 2016d
DBTD-CMP1	3 wt% Pt	20%TEOA	water	> 420 nm	4,600	3.3% at 400 nm	Wang et al., 2018
DBTD-CMP1	No	20%TEOA	water	>420 nm	2,460		Wang et al., 2018
PTEPB	No	No	water	>420 nm	218	10.3% at 420 nm	Zhang et al., 2017b
B-FOBT-1,3,5-E	No	10% TEOA	Water	>420 nm	9,600	1.8% at 420 nm
PTO-300	2.2 wt% Pt	10 v% TEOA	PBS buffer at pH 7	No	1,076		Schwinghammer et al., 2015
CTF-T1	1 wt% Pt	12.5 v% TEOA	water	>420 nm	380	2.4% at 420 nm	Bi et al., 2015
CTF-2	2.2 wt% Pt	20 v% TEOA	water	>420 nm	296	1.60%	Meier et al., 2017
CTP-2	3 wt% Pt	10 v% TEOA	water	>420 nm	500		Lan et al., 2018
CTF-1-100W	3 wt% Pt	10 v% TEOA+3 v% MeOH	water	>420 nm	5,500	6% at 420 nm	Xie et al., 2018
CTFS10	1 wt% Pt	10 v% TEOA	water	>420 nm	2,000		Li et al., 2016b
MoS_2_-CTF	1 wt% Pt	10 v% TEOA	water	>420 nm		
N3-COF	2.2 wt% Pt	1 v% TEOA	PBS buffer at pH 7	>420 nm	1,703	0.44% at 450 nm	Vyas et al., 2015
N2-COF	1.2 wt% Co	1 v% TEOA	pH 8 ACN/H2O 4:1	AM 1.5	782	0.16% at 400 nm	Banerjee et al., 2017
PTP-COF	2.2 wt% Pt	1 v% TEOA	PBS buffer at pH 7	AM 1.5	84		Haase et al., 2017
A-TEBPY-COF	2.2 wt% Pt	10 v% TEOA	PBS buffer at pH 7	AM 1.5	98		Stegbauer et al., 2018
TP-BDDA-COF	3 wt% Pt	10 vt% TEOA	water	>395nm	324		Pachfule et al., 2018
PyBT-2	No	0.01 M Co(NO_3_)_2_·6H_2_O	La_2_O_3_ pH buffer	>300 nm	579		Xu et al., 2018
PTEPB	No	No	water	>420 nm	110		Zhang et al., 2017b
CTF-T1	1 wt% RuO2	0.01 M AgNO_3_	La_2_O_3_ pH buffer	>420 nm	9		Bi et al., 2015
CTP-2	3 wt% Co	0.01 M AgNO_3_	La_2_O_3_ pH buffer	>420 nm	30		Lan et al., 2018
CTF-1-100W	3 wt% RuOx	0.2 M AgNO_3_	water	>420 nm	140	3.8% at 420 nm	Xie et al., 2018

*The results were concluded from the references*.

## Outlook

POPs have greatly enriched the photocatalyst family and synthetic methodologies as a promising alternative to state-of the-art metal photocatalysts, and competitive to the carbon nitrogen materials. Some basic principles for developing efficient POP photocatalysts for water splitting would be elucidated. Firstly, broad light absorbance and well-selected band gap (around 2~3 eV) allow effective utilization of the maximum portion of the visible light to facilitate HER and OER processes in the mesopores. Designation of rationally polarized skeleton and introduction of the functional group such as alkynyl are efficient approaches that aim to address harvest and utilization of visible-light. Secondly, atomic single-layer or few-layer ordered structures and more exposed active sites due to high surface areas ensure the high activities of POP photocatalysts, diverse topologies and morphologies emphasis on planarity conjugation and nanostructured crystalline remain to be pursued. Last, significant enhancements of photocatalytic activities would be expected via chemical modification on the backbone or composite metal active sites. Engineering hybrid photosystem is another pathway to the object of high efficiency water splitting, by fabricates heterojunction that combine two semiconductor which own different band gap (Tran et al., 2012). As organic semiconductor photocatalysts, combined the inorganic photocatalyst such as metal oxide that characterize lower overvoltage and higher current density (Osterloh, 2013; Ge et al., 2017) might offer a wealth of breakthrough on visible-light driven water splitting (Zhang et al., 2010b; Tian et al., 2015). However, combination of POP photocatalyst and metal oxides for photocatalytic water splitting still a nearly blank area. These remain mostly unexplored and should be pursued in the future. Overall, the emerged POP photocatalysts have shown considerable advantages including easily tunable morphology, porosity, chemical structure and energy band for photocatalytic water splitting. However, there are still some drawbacks for POPs in photocatalytic water splitting.

1. Most of photocatalytic water splitting process of POPs requires the presence of platinum, but for economic and sustainable developmental reason the efforts on exploring noble-metal-free photocatalytic water splitting of POPs are essential. Many pure CMPs are active in photocatalytic water splitting, but it is clear that residual palladium from the polymerization process has made a contribution. Besides, cheap and abundant cobalt has been used as co-catalyst in other POP photocatalytic systems, which is expected to instead of platinum. Sacrificial agent is commonly used to promote the decomposing of water. However, it is unrecyclable and pollutes environment.
2. Photocatalytic hydrogen production have been extensively investigated, while on contrary photocatalytic oxygen production remains stagnant. As water oxidation proceeds more difficultly in kinetic and energetic aspects, photocatalytic oxygen production is a more challenging study in the future. CTFs are demonstrated as candidate for highly efficient photocatalytic oxygen evolution. It can be envisaged that fast-developing synthetic strategies would offer CTF more expectant properties.
3. POPs possess considerable photocatalytic activity in water splitting, for all that, the apparent quantum yield is still lower compared with the carbon nitrogen materials (highest AQY at 60% under visible light irradiation of 420 nm) (Lin et al., 2016; Zhang et al., 2018). POPs are potentially tailored and optimized with high-performance for efficient water splitting, and functionally directed engineering on structure-properties is demanded.

In summary, POPs are promising and challenging for water splitting, this minireview would like to give a slight inspiration on design, synthesis and modulation of POPs in the field of photocatalytic water splitting. For competitive to state-of the-art metal photocatalysts and carbon nitrogen materials, more well-designed POPs with diverse building blocks, synthetic methods and functional groups are expected. Unlike the mature research of metal oxide photocatalysts, POPs are new emerged platform of water splitting. The drawbacks above impede the commercialization and industrialization of POP, more research of highly efficient photocatalytic water splitting system is still needed to challenge the practical requirements of energy and environment.

## Author Contributions

CX and WZ contributed equally to the work. All the authors listed have made contribution to drafting and writing the manuscript.

### Conflict of Interest Statement

The authors declare that the research was conducted in the absence of any commercial or financial relationships that could be construed as a potential conflict of interest.
